# Subthreshold Fear Conditioning Produces a Rapidly Developing Neural Mechanism that Primes Subsequent Learning

**DOI:** 10.1523/ENEURO.0113-19.2019

**Published:** 2019-06-27

**Authors:** Kehinde E. Cole, Jessica Lee, Michael Davis, Ryan G. Parsons

**Affiliations:** 1 Department of Psychology, Stony Brook University, Stony Brook, NY 11794; 2 Department of Psychiatry and Behavioral Sciences, Emory University, Atlanta, GA 30329

**Keywords:** amygdala, conditioning, fear, memory, metaplasticity, priming

## Abstract

Learning results in various forms of neuronal plasticity that provide a lasting representation of past events, and understanding the mechanisms supporting lasting memories has been a primary pursuit of the neurobiological study of memory. However, learning also alters the capacity for future learning, an observation that likely reflects its adaptive significance. In the laboratory, we can study this essential property of memory by assessing how prior experience alters the capacity for subsequent learning. Previous studies have indicated that while a single weak fear conditioning trial is insufficient to support long-term memory (LTM), it can facilitate future learning such that another trial delivered within a protracted time window results in a robust memory. Here, we sought to determine whether or not manipulating neural activity in the basolateral amygdala (BLA) using designer receptors exclusively activated by designer drugs (DREADDs) during or after the initial learning trial would affect the ability of the initial trial to facilitate subsequent learning. Our results show that inhibiting the BLA in rats prior to the first trial prevented the ability of that trial to facilitate learning when a second trial was presented the next day. Inhibition of the BLA immediately after the first trial using DREADDs was not effective, nor was pharmacological inhibition of protein kinase A (PKA) or the mitogen-activated protein kinase (MAPK). These findings indicate that the neural mechanisms that permit an initial subthreshold fear conditioning trial to alter later learning develop rapidly and do not appear to require a typical post-learning consolidation period.

## Significance Statement

Laboratory studies of memory are typically designed to identify mechanisms that allow the brain to represent past experience. The purpose of this ability is likely so that prior learning can direct future behavior, however studies are most often focused on the storage aspects of memory and less on how later experience is altered. We used a fear conditioning paradigm in which a single trial, subthreshold for producing long-term behavioral change, primes future learning when a second trial is later encountered. Neural activity in the amygdala was required for long-term memory (LTM) priming; however, post-training disruption of neural activity or cell signaling events had no effect. These results provide insight into the mechanisms that allow prior aversive experience to modify new learning.

## Introduction

One of the primary goals of the neurobiological study of learning and memory has been to identify how the brain stores prior experience. The typical approach has been to present a learning experience along with a manipulation designed to mimic or disrupt a particular neurobiological process and then to assess the effectiveness of the manipulation in a subsequent test session. This methodology has been extremely useful in determining how the brain represents past experience, and as a result we now understand a great deal about the neural mechanisms supporting memory storage (Josselyn et al., 2015; Tonegawa et al., 2015). It is also recognized that the brain’s ability to represent past experience allows prior experience to direct future behavior (Dudai, 2009; Schacter, 2012; Gershman, 2017; Parsons, 2018). The typical approach captures this, but only insomuch as behavioral performance at test reflects learning produced by the prior training event. What the typical approach to studying memory often fails to reveal is the extent to which past experience might alter future learning.

To address how prior learning affects subsequent experience a simple modification to most procedures can be made such that the testing phase is replaced with another training experience. In the last several years a number of reports have used this basic approach to determine how prior experience affects subsequent learning, and these studies have revealed that past experience has a profound effect on future learning (Hulme et al., 2013; Viola et al., 2014). For example, motivated by *in vitro* studies of synaptic tagging and capture, several studies have reported that relatively innocuous experiences that normally produce only transient memories can be transformed into robust long-term memory (LTM) if another experience had occurred near in time (Moncada and Viola, 2007; Ballarini et al., 2009; Moncada et al., 2011; Redondo and Morris, 2011). Other studies have reported similar findings, although on a much longer time scale. For example, modified Pavlovian fear conditioning procedures have been developed that allow for the study of how prior fear conditioning alters later learning. In one of these paradigms, a single pairing of light and mild shock that was not able to support memory formation alone, was able to prime subsequent learning such that a second identical trial presented an hour to several days later resulted in a robust fear memory (Parsons and Davis, 2012). Recent studies have reported that standard auditory fear conditioning is able to enhance later fear learning, even when the subsequent conditioning is not identical to the prior training (Rashid et al., 2016; Lee et al., 2018).

In the prior study demonstrating priming after a single fear conditioning trial, it was also reported that although a single trial did not support LTM, it did activate both protein kinase A (PKA) and extracellular signal-related mitogen-activated protein kinase (ERK/MAPK) signaling in the basolateral amygdala (BLA; Parsons and Davis, 2012). The activation of PKA by the first training trial was necessary for the priming effect because blocking PKA activity in the BLA during the first training trial prevented the ability of that trial to prime learning. In a subsequent study, it was reported that LTM formation following the second trial also required PKA signaling in the BLA (Parsons et al., 2016c). However, what is unclear is whether the requirement for neural activity and cell signaling events in the BLA change as a function of time following the initial priming event. The experiments reported here were designed to address this gap in knowledge.

Here, using the paradigm described above, we first tested whether interrupting neural activity in the BLA during or immediately after the first training trial affected the facilitation of later learning. We found, using the hM4Di designer receptor exclusively activated by designer drugs (DREADD) receptor to inhibit neural activity in the BLA, that the priming effect required neural activity in the BLA during, but not immediately after, the first training trial. Next, we delivered inhibitors of PKA and ERK/MAPK into the BLA shortly before or immediately after the initial training trial. We found that interrupting PKA or ERK/MAPK immediately after an initial trial did not affect the ability of the initial trial to prime learning to the second trial, whereas pre-training blockade of MAPK was effective in disrupting LTM formation. Collectively, our results indicate that the ability of an initial fear conditioning trial to facilitate subsequent learning depends on neural mechanisms that develop rapidly during the initial training trial.

## Materials and Methods

All procedures were conducted with approval from the Institutional Animal Care and Use Committee and in accordance with the National Institutes of Health guidelines for the care and use of laboratory animals

### Subjects

A total of 126 adult, male, Sprague Dawley rats obtained from Charles River Laboratories served as subjects. Rats were housed in pairs in a colony room maintained on a 12/12 h light/dark cycle, and food and water were provided freely throughout the experiment. On delivery, rats were left undisturbed for 7 d, and then each rat was gently handled for 5 min every day for 6 d. During the last 3 d of handling, rats were carted into the laboratory to acclimate them to being transported. Behavioral procedures began after the sixth day of handling.

### Surgical procedures

On the day of surgery, rats were anaesthetized with ketamine (87 mg/kg) and xylazine (10 mg/kg) or dexdomitor (0.5 mg/kg) and ketamine (75 mg/kg). For the DREADD experiments, rats were bilaterally injected with an adeno-associated viral (AAV) vector intracranially aimed at the BLA (AP = –3.2, L = ±5.1, V = –8.0). To do so, a 22-gauge cannula was lowered into place and an internal cannulae (28 gauge) was used to deliver the virus. For the pharmacological experiments, the rats were bilaterally implanted with a 22-gauge indwelling cannulae that was anchored to the skull using stainless screws and dental cement. Dummy cannulas were inserted into the guide cannulas to prevent blockage. After surgery, animals received subcutaneous injections of meloxicam (1 mg/kg) and glycopyrrolate (0.02 mg/kg).

### Drug preparation and infusion

The PKA inhibitor Rp-cAMPS (Tocris Bioscience) was diluted with saline to a concentration of 36 μg/μl. The MAPK inhibitor, U0126, was dissolved in 50% DMSO to a final concentration of 2 μg/μl. Rats received bilateral infusions of either Rp-cAMPS, U0126, saline, or 50% DMSO in saline (vehicle). Drugs were infused into the amygdala 30 min prior or immediately after the first training trial at a rate of 0.15 μl/min, with a total volume of 0.5 μl/side. After the infusion, the cannulas were left in place for additional 2 min to allow diffusion from the tip of the cannula. The dummy cannulas were then replaced and rats were returned to their home cage. To activate the DREADD receptor, clozapine-N-oxide (CNO) was delivered via intraperitoneal injection. CNO was provided by the National Institutes of Health Drug Supply Program and was prepared by dissolving in DMSO, sonicating for ∼2 min in a water bath, and diluting with sterile saline to a final concentration of 5 mg/ml. The vehicle working solution was 5% DMSO in sterile saline.

### Virus infusion

Rats were stereotaxically injected with 0.6 μl/side (0.15 μl/min) of AAV vectors expressing a modified form of the human muscarinic receptor M4, hM4Di (AAV8-CaMKII-hM4Di-mCherry), or a control virus (AAV8-CaMKII-eGFP) of the same promoter and serotype, into the BLA. Both viruses were ordered from Addgene and were gifts from Bryan Roth (Addgene viral prep #50477-AAV8, #50469-AAV8). An additional control group included rats that were infected with the AAV8-CaMKII-hM4Di-mCherry viral construct, but received an intraperitoneal injection of vehicle (5% DMSO with saline). For experiment 1, rats were injected with CNO 1 h before the first training session and for experiment 2, rats received CNO injection immediately after the first training session.

### Histology

Cannula verification in the amygdala was determined by anesthetizing animals with an intraperitoneal injection of fatal plus solution (100 mg/kg). Rats were then transcardially perfused with 10% PBS followed by 10% buffered formalin. The brains were removed and stored in a 30% sucrose-formalin solution for at least 48 h. The brains were then frozen and sectioned on a cryostat at 40-μm thickness and stained for Nissl. Sections were examined using light microscopy and cannulae placements were determined with the aid of a rat brain atlas (Swanson, 2004). To be considered an accurate placement, cannulae tips needed to be within or no further than 0.5 mm from the BLA and medial to the external capsule. Due to an error during the staining procedure, placements for five rats could not be matched to the subject numbers. All five had accurate BLA placements, and thus were included in the final analysis. For the chemogenetic experiments, the location of virus expression was assessed using fluorescence microscopy at 10× magnification.

### Apparatus

**Figure 1.**
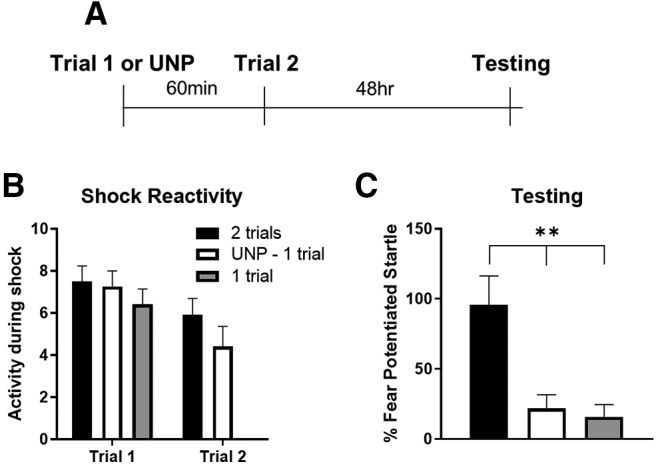
LTM priming requires paired presentation of light and shock. ***A***, Timeline of the experiment. Rats were with either a single paired trial (*N* = 10), two paired trials separated by 60 min (*N* = 13), or an unpaired trial followed by a paired trial (*N* = 11). ***B***, Shock reactivity did not differ between the groups. ***C***, Rats given two paired trials showed higher levels of fear-potentiated startle compared to both the unpaired-one trial and one trial groups; **p < 0.01 (Tukey HSD). Error bars, SEM.

Acoustic startle responses and fear learning were assessed by using a Startle Monitor II system (version 8.15, Kinder Scientific) or custom made Plexiglas and wire-mesh cages startle chambers (experiments with results in Figs. 1, 6), the details of which are described elsewhere (Parsons and Davis, 2012). The baseline startle and test sessions occurred in a set of four identical 17.5 × 9.2 × 7.5 cm restrainers. For the fear conditioning sessions, rats were placed in 26.67 × 20.96 × 15.9 cm (depth × width × height) Plexiglas and stainless-steel cages. The floor of these cages was made of stainless-steel bars through which shock could be delivered. Each of these sat atop load cell sensors and were housed individually within (40.64 × 40 × 49.53 cm) sound attenuating chambers. Movement of the restrainer produced by the startle response was detected by the load cell and transduced into a voltage change that was then converted to Newton. Speakers were located on the ceiling of each chamber through which a white noise burst was delivered (50 ms, 95 dB) to elicit the startle response. The same speakers produced a constant background noise of ∼52 dB. The light (4.0 s/82 lux) conditioned stimulus was delivered through an LED light panel positioned on the ceiling of the cabinets.

### Behavioral procedures

#### Baseline startle

**Figure 2.**
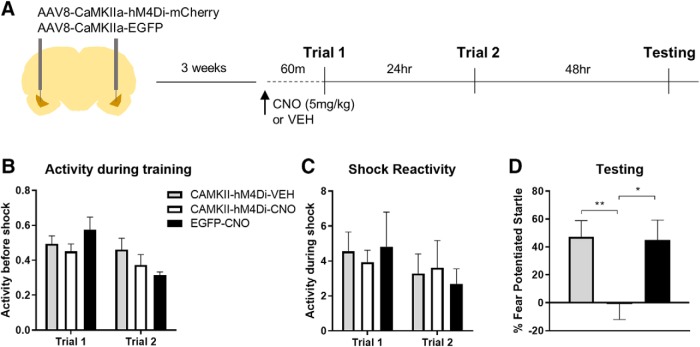
The ability of an initial fear conditioning trial to prime future learning depends on neural activity in the BLA. ***A***, Timeline of the experiment. Rats were infected with AAV8-CaMKIIa-hM4Di-mCherry or AAV8-CaMKIIa-EGFP targeting the BLA. Animals were given a single fear conditioning trial, 60 min before which they received an intraperitoneal injection of CNO or vehicle. A second trial was given the following day, and memory was tested 48 h later. ***B***, Activity during the baseline period of training did not differ between groups, nor did reactivity to the shock. ***C***, Rats expressing the hM4Di-DREADD receptor and given CNO (*N* = 7) before trial 1 showed significantly less fear potentiated startle compared to hM4Di rats given vehicle (*N* = 8) and compared to EGFP rats given CNO (*N* = 6). ***D***, **p < 0.01, *p < 0.05 (Tukey HSD). Error bars, SEM.

**Figure 3.**
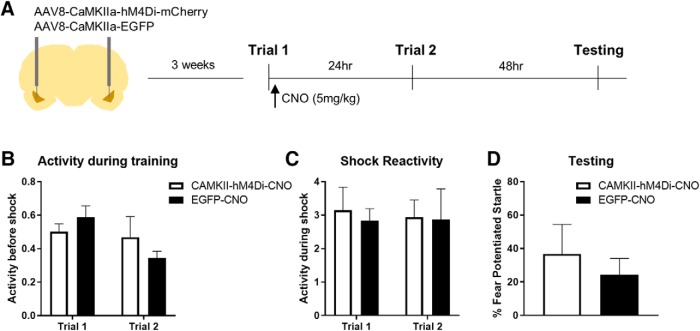
Neural activity in the BLA after the initial trial is not required for priming of future learning. ***A***, Timeline of the experiment. Rats were infected with AAV8-CaMKIIa-hM4Di-mCherry (*N* = 8) or AAV8-CaMKIIa-EGFP (*N* = 7) targeting the BLA. Rats were given a single fear conditioning immediately after which they received an intraperitoneal injection of CNO. A second trial was given the following day and memory was tested 48 h later. ***B***, Activity during the baseline period of training did not differ between groups, nor did reactivity to the shock (***C***) during the test session, and (***D***) levels of fear-potentiated startle did not differ in hM4Di and EGFP rats given CNO. Error bars, SEM.

**Figure 4.**
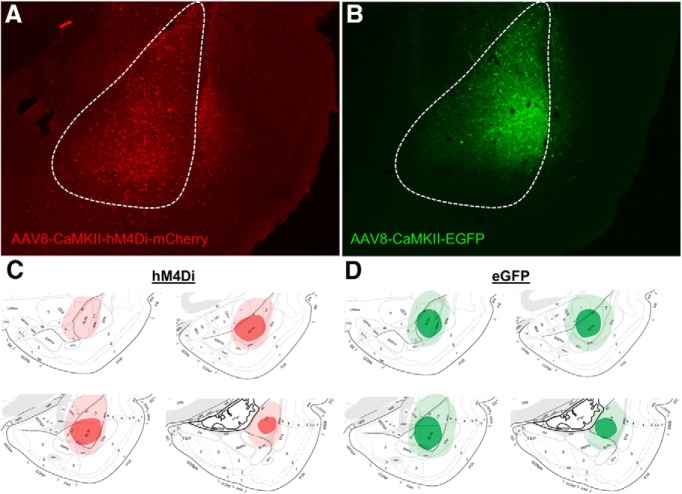
Photomicrographs depicting fluorescent labeling in the BLA in rats infected with AAV8-CaMKIIa-hM4Di-mCherry (***A***) or AAV8-CaMKIIa-EGFP (***B***), for the rats included in Figures 1, 2. Lighter shade of red and green indicate the maximal extent of viral expression for hM4di (***C***) and EGFP (***D***), respectively. Darker color shapes indicate the smallest spread extent of spread for the same viruses. Rat brain atlas images are from Swanson (2004).

**Figure 5.**
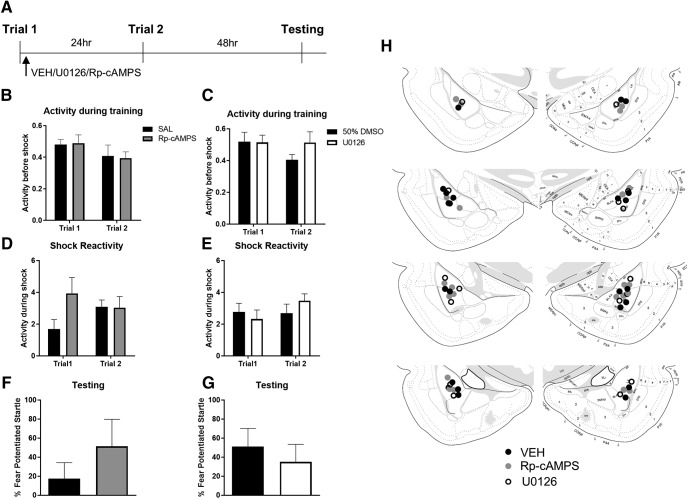
Pharmacological inhibition of PKA or MAPK immediately after trial 1 had no effect on the priming of future learning. ***A***, Timeline for the experiments. Rats were given infusions of the PKA inhibitor Rp-cAMPS (*N* = 8) or its vehicle (saline, *N* = 10), or the MAPK blocker U0126 (*N* = 8) or its vehicle (50% DMSO, *N* = 8), into the BLA immediately after the initial training trial. Activity during the baseline period of training did not differ between groups in either experiment (***B***, ***C***), nor did reactivity to the shock (***D***, ***E***). There were no differences in levels of fear-potentiated startle during the test session for either experiment (***F***, ***G***). Dots indicate cannula placements for the rats included in this experiment (***H***). Rat brain atlas images are from Swanson (2004). Error bars, SEM.

All subjects underwent baseline startle amplitude procedures on two consecutive days. After a 5-min acclimation time, rats were presented with 30, 95 dB, 50-ms white noise bursts, with a 30-s intertrial interval between each burst. Startle amplitude was defined as either the maximum change in Newtons that occurred during the first 500 ms after onset of the white noise burst (experiments indicated in Figs. 2–5), or by displacement of an accelerometer that produced a voltage output proportional to the velocity of cage movement during the first 200 ms after onset of the startle-eliciting noise burst. The mean startle amplitude across the 2 d were determined for each rat, and animals were assigned to groups that had equivalent startle amplitudes.

#### Fear conditioning

**Figure 6.**
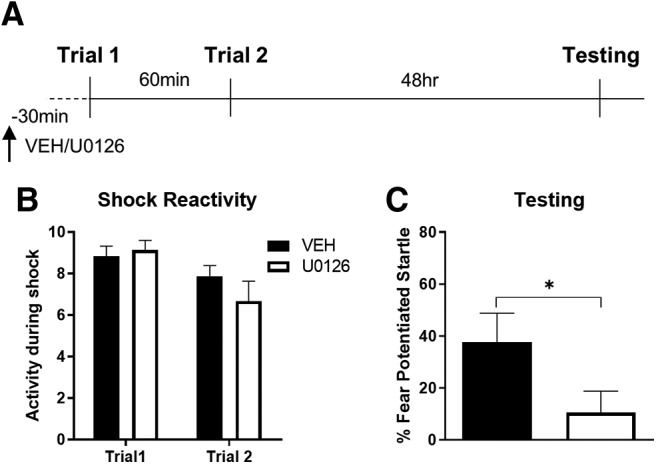
Pre-training inhibition of MAPK disrupted LTM formation. ***A***, Timeline for the experiment. Rats were given infusions of the MAPK inhibitor U0126 (*N* = 9) or its vehicle (*N* = 13) into the BLA 30 min before training. Reactivity to the shock on either the first or second trial did not differ between groups. ***B***, Rats given U0126 before training showed less fear-potentiated startle during the testing session. ***C***, *p < 0.05 (*t* test). Error bars, SEM.

The following day after baseline startle, rats were exposed to the fear conditioning procedure. In the first experiment (Fig. 1), rats were placed in shock cages and after 5 min separate groups were given a single light (4 s, 82 lux)-shock (0.5 s/0.4 mA) trial, two trials spaced by 60 min, or an unpaired presentation of light and shock followed 60 min later by a single paired trial. For the unpaired trial, a single shock was delivered 2 min before the light. For the experiments indicated in Figures 2–5, rats were placed into shock cages where, after a 5-min baseline period, they received a single pairing of light with a shock. In the experiments testing the effect of chemogenetic inhibition of the BLA, 1 h before the first conditioning trial, or immediately after, rats received an intraperitoneal injection of CNO or vehicle. In the experiment testing the effect of PKA and MAPK inhibitors, infusions were made into the BLA immediately after the first trial. All rats then received a second identical training trial 24 h later. Finally, in the experiment indicated in Figure 6, rats were given two paired trials separated by 60 min. Injections of U0126 were made 30 min before the beginning of the conditioning session.

#### Fear memory testing

For all experiments, 48 h after the final training session, rats were placed backed into the chamber used during the baseline acoustic startle sessions. After 5 min, they were presented with 30, 95-dB startle trials to habituate startle responses before the light startle trials. Rats then received 40 additional test trials, consisting of 10 light-startle trials, each followed by three startle-alone trials. For the light-startle trials, the 95-dB white noise burst was presented 3.5 s after onset of the cue. For the startle-alone trials, the 95-dB white noise burst was presented in the absence of the light.

### Data analysis

Fear-potentiated startle was calculated by finding the average of the 10 light-startle trials and of the 30 startle-alone trials that were presented between the light-startle trials. A difference score was computed by subtracting the startle alone trials from the light-startle trials, and these values were expressed as a percentage. Shock reactivity for both trials was measured in the same manner as acoustic startle (i.e., cage movement), as the peak change in force that occurred during the 0.5-s shock period. Activity during training was also calculated before administration of the shock, again by measuring the peak change in force that occurred every 30 s for 5 min before the shock. These data were averaged across the 5-min baseline period. For the experiment testing the effect of pharmacological inhibition of PKA and MAPK, we combined data across the vehicle groups because these two groups did not differ statistically (mean saline = 17.46, mean 50% DMSO = 51.08; *t*_(16)_ = –1.44, *p* = 0.08). Fear-potentiated startle data were analyzed using a one-way ANOVA test and Tukey HSD *post hoc* test where appropriate. Student’s *t* tests (one-tailed) were used to test for differences in experiments with two groups. Shock reactivity and activity values were analyzed using a repeated measures ANOVA with group as a between subject factor and trial as a within-subject factor. For all tests, *p* < 0.05 was considered significant.

## Results

### LTM priming requires paired presentation of light and shock

In the first experiment, we assessed the conditions that permit the antecedent event to facilitate subsequent learning. We compared rats given two paired trials, a group given an unpaired trial followed by a paired trial, and rats presented with only a single trial (Fig. 1*A*). An ANOVA on data from the initial training session showed no differences between groups on activity levels before shock (*F*_(2,31)_ = 0.263, *p* = 0.771; data not shown), and no differences in reactivity to the shock (*F*_(2,31)_ = 0.629, *p* = 0.540; Fig. 1*B*). Only two groups received a second shock, and a *t* test on these data showed no difference in shock reactivity in the rats given two paired trials versus those given an unpaired trial followed by a paired trial (*t*_(13)_ = 1.311, *p* = 0.203; Fig. 1*B*). Fear-potentiated startle scores during the test session (Fig. 1*C*) were compared using an ANOVA, which revealed a significant effect of group (*F*_(2,31)_ = 9.269, *p* = 0.001). Tukey HSD *post hoc* tests showed significant differences between those rats trained with two trials compared both to the one trial group (*p* < 0.01) and the unpaired-one trial group (*p* < 0.01). The results indicate that an unpaired trial does not prime LTM in the same manner as a paired trial, suggesting that this phenomenon is associative.

### Pre-training inhibition of the amygdala prevents LTM priming

Here, we tested whether or not the ability of an initial fear conditioning trial to prime subsequent learning depended on neural activity in the amygdala during the first training trial. Rats infected with either the inhibitory DREADD receptor hM4Di or rats expressing a control virus were given CNO injections 1 h before a single pairing of a light and foot shock. A third group of rats expressing hM4Di were given injections of the vehicle at the same time point. All rats were given a second trial the next day and fear memory was tested 48 h later (Fig. 2*A*). First, we examined whether activity levels before shock, or reactivity to shock, differed across groups. We used a repeated measures ANOVA with group as a between subject factor, and trial as a within subject factor. There was no effect of group (*F*_(2,17)_ = 0.635, *p* = 0.533), and no significant trial × group interaction (*F*_(2,17)_ = 3.285, *p* = 0.062). There was a significant effect of trial (*F*_(2,17)_ = 10.602, *p* < 0.01), presumably driven by the fact that activity levels were lower before the second shock (Fig. 2*B*). A repeated measures ANOVA on shock reactivity data revealed no effect of group (*F*_(2,17)_ = 0.009, *p* = 0.991), no effect of trial (*F*_(2,17)_ = 2.493, *p* = 0.133), and no group × trial interaction (*F*_(2,17)_ = 0.404, *p* = 0.674) during training, indicating that all rats had a relatively normal reaction to the foot shocks on both days (Fig. 2*C*). Next, we assessed whether hM4Di-mediated inhibition in the BLA before the first training trial prevented the priming of future learning. A one-way ANOVA on fear-potentiated data from the test session (Fig. 2*D*) revealed a significant effect of group (*F*_(2,17)_ = 6.954, *p* < 0.01). Tukey HSD *post hoc* tests revealed a significant difference between hM4Di-CNO and hM4Di-VEH-treated rats (*p* < 0.01), and hM4Di-CNO and EGFP-CNO (*p* < 0.05) rats. These results indicate that disrupting neural activity in the BLA before an initial training trial prevents the ability of that trial from priming learning to the second trial, and thus disrupts the formation of LTM.

### Post-training inhibition of the amygdala does not affect LTM priming

Next, we tested whether inhibiting amygdala activity immediately after the first training trial would prevent the priming of LTM when a second trial is presented the next day. Rats infected with a virus expressing hM4Di or controls received an intraperitoneal injection of CNO immediately after the first training trial. All rats were given a second trial the next day and fear memory was tested 48 h later (Fig. 3*A*). First, we examined whether activity levels before shock, or reactivity to shock, differed across groups. We used a repeated measures ANOVA with group as a between subject factor, and trial as a within subject factor. There was no effect of group (*F*_(1,13)_ = 0.672, *p* = 0.427), no effect of trial (*F*_(1,13)_ = 2.799, *p* = 0.118), and no interaction (*F*_(1,13)_ = 0.049, *p* = 0.829). These data indicate that activity levels between groups were similar on both trial days and that activation of hM4Di after the first trial did not have a delayed effect on baseline activity before the second trial (Fig. 3*B*). Next, we performed similar analyses on the shock reactivity data. There were no significant differences between group (*F*_(1,13)_ = 0.010, *p* = 0.924), no effect of trial (*F*_(1,13)_ = 0.000, *p* = 0.989), and no interaction (*F*_(1,13)_ = 0.100, *p* = 0.757), again indicating that shock reactivity was similar between groups and the immediate post-trial 1 activation of hM4Di did not affect reactivity to shock the next day (Fig. 3*C*). Finally, we tested whether inhibition of BLA immediately after the initial trial would prevent the ability of that trial from priming LTM when a second trial follows the next day. A *t* test on fear-potentiated startle data from the testing session showed that rats expressing hM4Di or EGFP and receiving post training injections of CNO after the first training trial showed no difference between the two groups (*t*_(13)_ = –0.624, *p* = 0.271). Thus, although pre-training inhibition of the BLA prevented the first trial from priming subsequent learning, in contrast, inactivation of the amygdala after the first training trial did not. These data suggest that the process necessary for the first training trial to prime LTM develops rapidly. Figure 4 shows the extent of viral expression in the amygdala in rats receiving hM4Di-mCherry or EGFP.

### Post-training blockade of MAPK or PKA does not affect LTM priming

In this experiment, we used pharmacological agents that have a more established effect on memory consolidation when delivered into the BLA (Schafe and Ledoux, 2000; Schafe et al., 2000). Rats were given infusions of the MAPK inhibitor U0126, the PKA inhibitor Rp-cAMPS, or their vehicles, immediately after the first training trial, using conditions in which infusion of these compounds into the BLA before trial 1 did block priming (Parsons and Davis, 2012; Parsons et al., 2016c). All rats were given a second trial the next day and fear memory was tested 48 h later (Fig. 5*A*). First, we used a repeated measures ANOVA to test for differences between groups and across trials in activity levels before shock during training. When comparing rats given Rp-cAMPS to controls given saline, the analyses revealed no effect of group (*F*_(1,16)_ = 0.087, *p* = 0.771), no effect of trial (*F*_(1,16)_ = 2.105, *p* = 0.166), and no interaction (*F*_(1,16)_ = 0.001, *p* = 0.972; Fig. 5*B*). An identical analysis was performed on data from rats given U0126 or 50% DMSO (Fig. 5*C*), again showing no effect of group (*F*_(1,14)_ = 0.979, *p* = 0.339), no effect of trial (*F*_(1,14)_ = 1.403, *p* = 0.256), and no interaction (*F*_(1,14)_ = 1.360, *p* = 0.236). A similar analysis on the shock reactivity was performed from both experiments. In rats given saline or Rp-cAMPS (Fig. 5*D*), there was no effect group (*F*_(1,16)_ = 3.331, *p* = 0.087), no effect of trial (*F*_(1,16)_ = 0.080, *p* = 0.781), and no interaction (*F*_(1,16)_ = 2.846, *p* = 0.111). Similarly, when comparing rats given 50% DMSO to those given U0126 (Fig. 5*E*), there was no effect of group (*F*_(1,14)_ = 0.109, *p* = 0.746), no effect of trial (*F*_(1,14)_ = 1.290, *p* = 0.275), and no interaction (*F*_(1,14)_ = 1.634, *p* = 0.222). Finally, we used *t* tests to compare fear-potentiated startle during the test session for both experiments (Fig. 5*F*,*G*). There was no significant difference when comparing the saline and Rp-cAMPS groups (*t*_(16)_ = 1.112, *p* = 0.141), and no difference between the 50% DMSO and U0126-treated rats (*t*_(14)_ = 0.646, *p* = 0.264). These data suggest that the mechanisms that allow for a single light shock pairing to alter subsequent learning do not require cell signaling events generated immediately after the first trial.

### Pre-training blockade of MAPK disrupts LTM formation

To be sure the lack of effect of U0126 given after the first training trial in the prior experiment was not because there was something wrong with the drug, or other conditions, we tested the effect the MAPK inhibitor U0126 delivered into the BLA before training in which two trials were spaced by 60 min (Fig. 6*A*). First, we used a *t* test to assess whether the two groups differed in activity levels before the first training trial. Results from this comparison indicated no difference between groups (*t*_(20)_ = 1.601, *p* = 0.125; data not shown). Next, a repeated-measures ANOVA was used to test for differences between groups and across trials in shock reactivity. There were no differences between groups (*F*_(1,20)_ = 0.479, *p* = 0.497), no interaction (*F*_(1,20)_ = 1.793, *p* = 0.196), but a significant effect of trial (*F*_(1,20)_ = 9.379, *p* = 0.006) driven by lower shock reactivity on the second trial (Fig. 6*B*). Finally, we used a *t* test to compare fear-potentiated startle between the two groups during the test session. Results from this comparison showed a significant difference between groups, with the rats given U0126 showing lower fear-potentiated startle (*t*_(20)_ = 1.817, *p* = 0.04) than control rats (Fig. 6*C*). These data indicate that MAPK signaling in the BLA is required for LTM memory formation.

## Discussion

Prior studies have showed that fear learning can facilitate future learning when additional training is given at later time points (Rashid et al., 2016; Lee et al., 2018). Such facilitation effects are observed even when an amount of training is given that is subthreshold for producing LTM (Parsons and Davis, 2012), as is the case here. The present results also indicate that neural activity in the BLA during the initial training trial is required for facilitation of LTM on presentation of a second identical trial the next day. However, interfering with neural activity in the BLA immediately after the first trial did not prevent the ability of the first trial to prime LTM. We also tested whether or not interfering with ERK/MAPK or PKA signaling in the BLA immediately after the first trial would affect facilitation. Both of these cell signaling mechanisms are known to be necessary for the consolidation of fear learning (Abel et al., 1997; Bourtchouladze et al., 1998), and this includes studies showing that blocking their activity in the BLA prevents fear memory consolidation (Schafe and Ledoux, 2000; Schafe et al., 2000). However, we found that interfering with their activity in the BLA immediately after the first trial did not affect the ability of the first trial to prime learning. Together, our findings suggest that the neural mechanism that allows the initial experience to prime future learning develops rapidly during the antecedent learning event.

The identity of the specific cellular mechanisms supporting the facilitation of subsequent learning are unclear, however there are several plausible possibilities. First, the single training trial in our paradigm may result in rapid enhancement of neuronal excitability in the BLA. Consistent with this possibility, learning-related neural excitability changes in the BLA have been reported to emerge early on during conditioning (Rosenkranz and Grace, 2002) and are apparent immediately after fear learning (Sehgal et al., 2014). The case that learning-dependent changes in excitability underlie the facilitation of later learning is made stronger by the fact that cell signaling mechanisms we know to be engaged in the BLA after the first training trial (i.e., PKA and MAPK) are also known to be critical for the learning-related changes in excitability (Cohen-Matsliah et al., 2007; Oh et al., 2009). Moreover, learning-related changes in neural excitability involve the modification of ion channel function (Zhang and Linden, 2003; Disterhoft and Oh, 2006; Mozzachiodi and Byrne, 2010), and some of these are also known to be controlled by PKA and MAPK activity (Schrader et al., 2006; Hammond et al., 2008). However, whether the learning-related priming we see following a single trial is dependent on rapid alterations in intrinsic excitability is unknown, as is the presence and identity of specific modifications in ion channel function. Another possibility is that the initial learning experience engages neuromodulatory systems governed by norepinephrine (NE), and that subsequent learning is primed by virtue of their activity. Several prior observations make this a reasonable possibility including that: exogenous delivery of NE primes future contextual fear learning (Hu et al., 2007); interfering with NE signaling in the BLA during, but not after, fear conditioning prevents memory formation (Bush et al., 2010); and molecular signaling events downstream of NE (Schiff et al., 2017) are activated by a single trial in the paradigm employed here (Parsons and Davis, 2012).

The results we obtained using the inhibitory DREADD receptor hM4Di to inhibit neural activity in the BLA are similar to what has been reported using pharmacological inhibition of the BLA. Muscimol, a GABA agonist, has been shown to be effective in disrupting fear learning when given into the BLA during conditioning (Helmstetter and Bellgowan, 1994; Muller et al., 1997), however when applied immediately after fear conditioning it is not effective in blocking the consolidation of fear memory (Wilensky et al., 1999). The results are similar despite a different mechanism of action for hM4Di, which inhibits neuronal activity by activating inwardly rectifying potassium channels (Armbruster et al., 2007) and by interfering with presynaptic neurotransmitter release (Stachniak et al., 2014). The hM4Di-DREADD receptor is coupled to the Gi alpha protein, thus on CNO binding, the activity of adenylyl cyclase is reduced and cAMP-PKA signaling would be prevented. Given the necessity of this signaling pathway in memory consolidation, it is surprising then that we did not observe any effect when we injected CNO into hM4Di-expressing rats immediately after the first training trial. Although DREADD-mediated inhibition has been primarily used to block neural activity, its effects on intracellular signaling mechanisms suggest that it might target memory consolidation processes. In fact, at least one study has showed that activation of the hM4Di-DREADD receptor can disrupt memory consolidation when CNO is delivered after training (Zhu et al., 2014). Thus, our tentative conclusion for the DREADD experiments was that the priming effect after a single trial developed rapidly and did not undergo a typical consolidation period. Because DREADDs have not been widely used to target consolidation processes, we also tested the effect of blocking key cell signaling mechanisms after the first trial. Although post-training silencing of BLA appears not to affect memory consolidation, a number of studies have reported that interrupting the function of other neurobiological substrates in the BLA immediately after training does disrupt the consolidation of fear memory (Schafe and Ledoux, 2000; Schafe et al., 2000, 2005; Parsons et al., 2006a, b; Kwapis et al., 2011; Jarome et al., 2011). Thus, we tested whether inhibitors of PKA or MAPK delivered to the BLA would affect consolidation of fear memory. The fact that we also did not observe a disruption when pharmacological inhibitors of PKA or MAPK were delivered to the BLA immediately after trial 1 lends further support to the conclusion that the memory priming effect of the first trial does not have a typical consolidation process.

The effects of chemogenetic inhibition of BLA on learning in this report do not appear to be the result of off-target effects of CNO or an impairment of sensorimotor capabilities produced by inhibition of neural activity in the BLA. There is evidence that for peripheral delivery of CNO to activate DREADD receptors expressed in the central system, it must be metabolized and converted to clozapine (Gomez et al., 2017). There is some indication that CNO can have effects that are independent of designer receptor expression (MacLaren et al., 2016). However, we did not observe any effect of CNO on learning in rats expressing a control virus, suggesting that the effects on learning in the hM4Di-expressing rats were the result of activation of the DREADD receptor. In addition, CNO administration before training did not produce evidence of sensory or motor impairments because there was no difference in activity levels at the start of training, and there was no difference in shock reactivity in rats treated with CNO as compared to vehicle-injected rats.

In addition to identifying the specific molecular mechanisms in BLA that permit the priming effect, going forward we are also interested in defining the neural circuits activated by the initial training trial and that are necessary for the facilitation of learning, and whether when the second trial is presented the next day, the same circuits are engaged and necessary for LTM formation.

There are some existing data that may speak to the neural circuits involved in LTM memory priming. First, sensory thalamic and cortical input to the BLA relay a representation of the CS (Romanski and Ledoux, 1992; Shi and Davis, 2001; Kim and Cho, 2017). There is some indication that the duration of plasticity following fear learning might differ in thalamo-amygdala and cortico-amygdala pathways, in that LTP in the lateral amygdala induced by stimulation of the cortical pathway lasts 24 h, but not 3 d, whereas LTP induced via stimulation of the thalamic pathway lasts longer (Doyère et al., 2003). It would be of interest to test the contribution of these pathways, especially considering the time course of the priming effect (Parsons and Davis, 2012) is roughly in line with the duration of cortico-amygdala LTP. Second, as noted, NE release in the BLA during training is required for fear memory formation, but is not required immediately after fear conditioning. This suggests that our priming effect, which is also insensitive to post-training manipulations, might involve release of NE in the BLA via inputs from the locus coeruleus (LC). A recent study showed that LC to BLA inputs are involved in the acquisition of fear with a typical fear conditioning procedure (Uematsu et al., 2017).

In conclusion, the present results indicate that the ability of an initial fear conditioning trial to alter later learning depends on a rapidly developing neural mechanism in the amygdala. This conclusion is based on the observation that while interfering with neural activity, or cell signaling mechanisms in the BLA during the initial trial prevented that trial from facilitating learning to a second trial, the same treatments had no effect when applied immediately after. The approach employed here, in which two fear conditioning trials separated in time can be studied; the first, for its ability to prime subsequent learning, and the second, for its ability to produce LTM, is advantageous for understanding how prior experience alters the capacity for subsequent learning.

## Synthesis

Reviewing Editor: Mark Baxter, Mount Sinai School of Medicine Decisions are customarily a result of the Reviewing Editor and the peer reviewers coming together and discussing their recommendations until a consensus is reached. When revisions are invited, a fact-based synthesis statement explaining their decision and outlining what is needed to prepare a revision will be listed below. The following reviewer(s) agreed to reveal their identity: Hongjoo Lee, K. Matthew Lattal.

- The authors added additional experiments to address some comments raised. They chose 60 min interval between two trials instead of 24 hr interval used in their initial experiments. Perhaps they chose 60 min interval based on the findings from Parsons and Davis, 2012. Regardless, they should explain the rationale behind their decision. For example, I find it puzzling that they chose this 60 min interval even with MAPK inhibition study (Fig 6). According to Parsons and Davis (2012), MAPK was phosphorylated 60 min, but not 24 hr, after the first trial, and interestingly MAPK phosphorylation was only seen after 1 trial but not after 2 trials. Therefore, it would seem to make more sense to wait 24 hr to administer the second trial when the intended target was MAPK.

- Also, how long does U0126 last once infused into the BLA? Is it possible that MAPK is still inhibited by the time the second trial is given?

- Please separate the vehicles in the figure and report the DMSO along side the U0126 and saline along Rp-cAMPS.
